# Impact of instrumentation in lumbar spinal fusion in elderly patients

**DOI:** 10.3109/17453670903170505

**Published:** 2009-08-01

**Authors:** Thomas Andersen, Finn B Christensen, Bent Niedermann, Peter Helmig, Kristian Høy, Ebbe S Hansen, Cody Bünger

**Affiliations:** Spine Unit, Department of Orthopaedics E, Aarhus University HospitalAarhusDenmark

## Abstract

**Background and purpose** An increasing number of lumbar fusions are performed using allograft to avoid donor-site pain. In elderly patients, fusion potential is reduced and the patient may need supplementary stability to achieve a solid fusion if allograft is used. We investigated the effect of instrumentation in lumbar spinal fusion performed with fresh frozen allograft in elderly patients.

**Methods** 94 patients, mean age 70 (60–88) years, who underwent posterolateral spinal fusion either non-instrumented (51 patients) or instrumented (43 patients) were followed for 2–7 years. Functional outcome was assessed with the Dallas pain questionnaire (DPQ), the low back pain rating scale pain index (LBPRS), and SF-36. Fusion was assessed using plain radiographs.

**Results** Instrumented patients had statistically significantly better outcome scores in 6 of 7 parameters. Fusion rate was higher in the instrumented group (81% vs. 68%, p = 0.1). Solid fusion was associated with a better functional outcome at follow-up (significant in 2 of 7 parameters). 15 patients (6 in the non-instrumented group and 9 in the instrumented group) had repeated lumbar surgery after their initial fusion procedure. Functional outcome was poorer in the group with additional spine surgeries (significant in 4 of 7 parameters).

**Interpretation** Superior outcomes after lumbar spinal fusion in elderly patients can be achieved by use of instrumentation in selected patients. Outcome was better in patients in which a solid fusion was obtained. Instrumentation was associated with a larger number of additional surgeries, which resulted in a lesser degree of improvement. Instrumentation should not be discarded just because of the age of the patient.

## Introduction

Instrumented spinal fusion in elderly patients has been problematized due to the risk of screw loosening and comorbidity (Hu 1997, Okuyama et al. 2001, Okuda et al. 2006, Cho et al. 2007), leaving uninstrumented fusion as an obvious alternative. Spinal instrumentation has only been shown to increase fusion rates compared to uninstrumented fusion; the effect on functional outcome has been insignificant (Gibson and Waddell 2005). These studies have, however, all been performed using autograft as fusion material and also mostly in younger patients. The use of autograft has been problematized due to increased concern about the amount and extent of pain arising from the donor site (Arrington et al. 1996, Robertson and Wray 2001, Sasso et al. 2005). In order to overcome the problems of donor site pain, allograft and bone substitutes have gained increasing interest (Sandhu et al. 1999, Ehrler and  Vaccaro 2000). Fresh frozen allograft has been one of the most widely used. One randomized study has shown that it gives similar outcomes compared to autograft in instrumented posterolateral fusion (Gibson et al. 2002); however, most surgeons still believe that autograft has superior qualities (An et al. 1995, Sandhu et al. 1999, Ehrler and Vaccaro 2000). The use of allografts in the older patient population may be problematic due to reduced fusion potential (Lohmann et al. 2001, Laursen et al. 2003). One long-term study in an elderly population has shown significantly better outcomes in patients in which a solid fusion was obtained compared to those without (Kornblum et al. 2004). Thus, the beneficial effect of instrumentation in lumbar spinal fusion might be more pronounced in an older patient population fused with allograft.

The aim of this study was to compare instrumented and non-instrumented lumbar spinal fusion performed using fresh frozen allograft in patients older than 60 years with regard to functional outcome and fusion rates.

## Patients and methods

### Patient population

The study included all 94 patients (60 women), aged 60 years or older, who underwent a primary lumbar fusion using fresh frozen allograft at our department in the period from January 2001 through December 2005. The mean age was 70 (60–88) years (Table). They all underwent posterolateral spinal fusion, either as a non-instrumented procedure (51 patients) or as an instrumented procedure using either CD-Horizon (Medtronic Sofamor Danek, Minneapolis, MN) (31 patients) or TSRH pedicle screw systems (Medtronic Sofamor Danek) (12 patients). The allograft used was a fresh frozen femoral head. Main indications for fusion were spinal stenosis surgery where fusion was deemed necessary due to instability or the need for extensive decompression, or a high degree of back pain. 91 patients had decompression performed together with the fusion. Central laminectomy was performed in 73 patients; 18 had a laminotomy and 3 patients were fused without decompression of neural structures because of primarily back pain. Of the 8 patients in the non-instrumented group who had previous spine surgery, this was discectomy in 4 patients and partial laminectomy in the other 4 patients. In the instrumented group the previous surgeries consisted of 8 discectomies, 5 partial laminectomies, and 1 fusion of the sacroiliac joints.

**Table:** Characteristics of the study population and drop-outs according to treatment group.

	Follow-up population			Drop-outs		
	Instrumented	Non-instrumented	P-value	Instrumented	Non-instrumented	P-value
Sex (female/male)	26/9	23/13	0.3	3/5	8/7	0.5
Age at operation (years)	67 (65–68)	70.3 (68.3–72.4)	0.007	68 (63–72)	76 (73–80)	0.008
Age at follow-up (years)	71 (69–73)	74.3 (72.3–76.4)	0.03	72 (68–77)	80.3 (77–83)	0.005
Follow-up time (years)	4.7 (4.3–5.2)	4.0 (3.5–4.4)	0.02	4.5 (3.4–5.6)	3.9 (3.1–4.8)	0.3
Operation time (min)	222 (191–254)	154 (136–172)	<0.001	215 (149–280)	179 (154–205)	0.4
Blood loss (mL)	898 (558–1238)	615 (413–816)	0.1	1012 (-97–2121)	672 (375–968)	0.9
Hospitalization (days)	13 (11–14)	13 (11–15)	0.3	14 (10–17)	12 (10–14)	0.4
Diagnosis			0.4			0.2
Degenerative	1	3		0	0	
Stenosis	14	19		4	11	
Stenosis + deg. olisthesis	11	9		1	3	
Stenosis + deg. scoliosis	9	5		3	1	
Operated level(s)			0.4			0.5
1 level	8	12		2	6	
2 levels	14	14		2	5	
3 levels	7	8		3	4	
4 + 5 levels	6	2		1	0	
Additional neural decompression			0.7			
None	1	2		0	0	
Laminotomy	8	10		0	0	
Laminectomy	26	24		8	15	
Previous spine surgery	9	7	0.5	5	1	0.004
Radiographic fusion at last follow-up	29 (83%)	26 (74%)	0.3	6 (75%)	8 (53%)	0.3

Values are mean (95% CI) or number (%).

All patients who were still alive were mailed the questionnaires described below in November 2007 to assess their functional outcome and quality of life at this follow-up. After 1 month, a written reminder was sent and no further contact was made after this.

Of the 94 patients, 4 patients had died at the time of the follow-up, 71 patients responded with completed questionnaires, and 3 patients stated that they felt unable to complete the questionnaires, 1 because of dementia and 2 because of other significant comorbidities. 16 patients did not respond at all. Thus, available response rate was 74/90 (82%). This resulted in an overall follow-up rate of 76% and an available follow-up rate of 79%. Average length of follow-up was 4.3 years with slightly longer follow-up time in the instrumented group (Table). Characteristics of the drop-outs are given in the Table. Twelve patients missed answering the subjective evaluation question and 4 patients missed listing their medication use. One patient in the non-instrumented group missed her follow-ups at 1 and 2 years and had no radiographs performed.

### Outcome parameters

Functional outcome was assessed by the Dallas pain questionnaire (DPQ). The DPQ assesses the impact of chronic spinal pain in 4 categories: Daily activities, Work-Leisure activities, Anxiety-Depression and Social interest. A high score indicates a high influence of back pain on the daily life of the patient and thus a poor outcome (Lawlis et al. 1989). Back and leg pain was measured using the pain assessment index from the low back pain rating scale (LBPRS). It comprises 3 scales each for back and leg pain (pain now, worst and average pain in the last 14 days), which are added to give a response scale ranging from 0 to 60 (Manniche et al. 1994).

Both the DPQ and the LBPRS pain index were completed preoperatively, at the 1- and 2-year follow-up, and at the mailed follow-up. Furthermore, the patients who answered the mailed follow-up completed the SF-36 generic health survey measure (Ware 2000). As subjective global evaluation, the patient's answer to the question “Now that you know the result, would you undergo the procedure again?” was used. This question was also asked in the mailed intermediate follow-up questionnaire.

Fusion was assessed by the surgeon at 1- and 2-year follow-up using plain anteroposterior and lateral radiographs and the criteria suggested by Christensen et al. (2001).

Patients were asked to list their pain medication on a separate page in the questionnaire. Doses were summarized using defined daily dose (DDD) (www.whocc.no). The DDD is the assumed average maintenance dose per day for a drug used for its main indication in adults. Furthermore, drug use was classified as no use, occasional use, or daily use.

### Statistics

All data were analyzed using non-parametric statistics. Between-group comparisons of continuous variables were done using the Mann-Whitney rank-sum test for unpaired data or the Kruskal-Wallis test for equality of groups, when comparing more than 2 groups (without correction for ties). Significance of proportions was calculated using χ^2^-test. Significance level was 5% using two-tailed testing. Results are presented as mean (95% CI) unless otherwise stated. Intercooled Stata version 9.2 for Windows was the software used for the statistical analysis.

## Results

**Figure 1.**
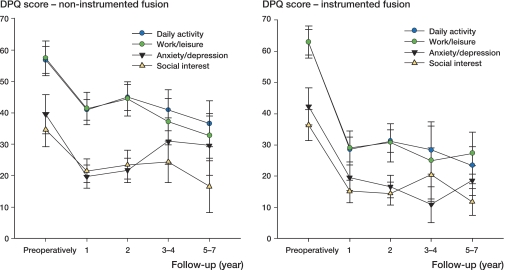
Dallas pain questionnaire (DPQ) scores according to follow-up time point in the 2 study groups.

**Figure 2.**
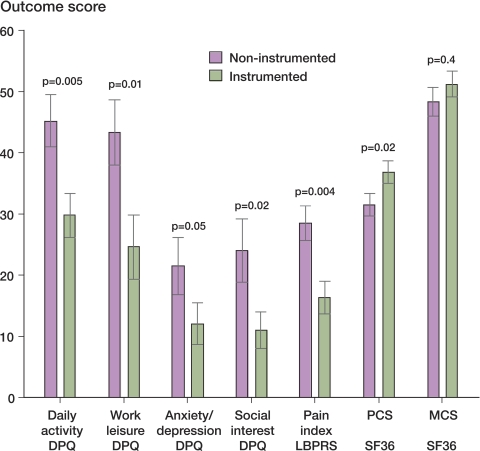
Long-term follow-up scores for all outcome parameters according to study group. PCS: Physical component summary scale; MCS: Mental component summary scale.

Both the non-instrumented and instrumented group improved from preoperatively to 1-year follow-up, with a much smaller improvement after that. With respect to the two activity-based DPQ scores, improvement was greatest in the instrumented group (Figure 1). Outcome was better in the instrumented group in all outcome parameters and reached statistical significance in 6 of 7 parameters (Figure 2). The SF-36 subscale outcome was still better in the instrumented group, although it was only statistically significant in the Bodily Pain (BP) category: 56 (48–65) vs. 41 (33–50) (p = 0.01). Controlling for differences in age and sex between the groups by using norm-adjusted scores of the SF-36, results were similar. Using the norm-adjusted score, there was a statistically significant difference in favor of the instrumented group in the BP subscale, 75 (64-87) vs. 56 (45-67) (p = 0.01), and in the physical component summary (PCS) scale, 85 (76-93) vs. 72 (64-81) (p = 0.02).

With respect to subjective evaluation at the mailed follow-up, 20/29 patients were positive in the instrumented group as compared to 14/30 in the non-instrumented group (p = 0.083). There was no difference between the groups with respect to use of pain medication. It was used on a daily basis by 20/35 and 18/32 in the non-instrumented and instrumented groups, respectively. Doses were slightly higher in the non-instrumented group, with a median DDD of 0.7 (0.1–1.33) as compared to 0.3 (0.1–1.0) in the instrumented group (p = 0.9).

Fusion rate was higher in the instrumented group with 35/43 patients fused as compared to 34/50 in the non-instrumented group (p = 0.1). Solid fusion was associated with a better functional outcome at long-term follow-up, although it only reached statistical significance in DPQ Daily activity, 34 (27–40) vs. 53 (42–65) (p = 0.007), and SF-36 PCS, 36 (33–39) vs. 29 (24–33) (p = 0.03), and almost in the LBPRS, 21 (16–26) vs. 25 (17–33) (p = 0.08). The non-union group had a higher use of analgesics with a median DDD of 1.0 (0.14–2.0) as compared to 0.3 (0.1–1.0) in the fused group (p = 0.3).

**Figure 3.**
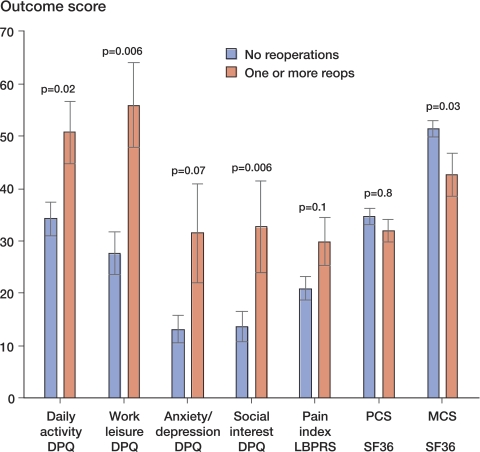
Outcome score at long-term follow-up according to whether the patient had additional spine surgery after the initial fusion procedure.

15 patients (6/51 in the non-instrumented group and 9/43 in the instrumented group) had a self-reported or chart history of repeated surgery to their lumbar spine after their initial fusion procedure: additional decompression at either adjacent or included levels, or re-fusions. 4 of the instrumented patients had the hardware removed due to loosening. Average age at operation in the reoperated group was 69 (60–79) years, which was no different from the group without reoperations. Functional outcome in the reoperated group was poorer than in single-surgery group (Figure 3). 8 of the reoperated patients reported use of pain medication on a daily basis, as compared to 30 in the group that did not have repeat surgery (p = 0.4). Furthermore, the median DDD was 1.4 (0.1–2.0) in the reoperated group as compared to 0.3 (0.1–1.0) in the single-surgery group (p = 0.3).

Comparing drop-outs from the the scores of their last follow-up, drop-outs in the non-instrumented group had poorer scores than the drop-outs in the instrumented group, but not statistically significantly so. Drop-out patients in both groups had poorer work/leisure scores when comparing drop-outs with full follow-up patients in the two groups; this was most pronounced in the non-instrumented group. Drop-out patients in the instrumented group generally had poorer anxiety/depression and social interest scores than their full follow-up counterparts. Inclusion of the scores of the last follow-up of drop-out patients in the group comparison did not change the results with regard to the significance of differences in the DPQ and LBPRS scales; nor did the overall significance of differences in the fusion status and the reoperation comparisons change when drop-out patients were included, except for the DPQ work-leisure score in the latter, which no longer reached significance (p = 0.1).

## Discussion

We found functional outcome to be better in older patients who were operated with instrumented fusion as compared to non-instrumented fusion. As it was not a randomized study, several possibilities for bias exist. Patients in the instrumented group were younger and had a slightly longer follow-up. However, after controlling for age and sex by adjusting with norm scores, the SF-36 results were still in favor of the instrumented group; nor did analyzing outcome scores according to length of follow-up change the fact that the results were better in the instrumented group. Furthermore, inclusion of drop-out patients in the comparisons did not change the results with respect to differences seen between the two groups. Thus, the likelihood of bias introduced by differences in drop-out rate appears to be small. Age was the only demographic variable that differed between the two groups, and it could not explain the differences in SF-36 scores observed. Despite this, it might still represent selection of patients for the instrumented procedure and selection bias favoring instrumentation cannot be ruled out entirely. In general, however, the results obtained in both patient groups are similar to what has been published previously (Glassman et al. 2007, Rampersaud et al. 2008). Thus, the differences cannot be explained by the non-instrumented group being exceptionally poor.

The randomized studies comparing fusion with and without additional pedicle screw fixation have mainly been performed in patient samples with an average age well below that of this series. One exception is the study by Fischgrund et al. (1997) in which the average ages in the instrumented and non-instrumented groups were 69 and 66 years, respectively. In the original study there was a statistically significant difference in fusion rates, but not in patient-assessed outcome. In a later long-term follow-up on this study, Kornblum et al. (2004) compared patients with solid arthrodesis to those with pseudoarthrosis and could demonstrate better outcome in those who were solid-fused. They interpreted this in favor of instrumentation, although the study involved a smaller patient material than the original and the data were not analyzed according to the original assigned treatment groups. In a cohort study comparing laminectomy alone to laminectomy with non-instrumented or instrumented fusion, Katz et al. (1997) could not demonstrate any beneficial effect of instrumentation (relative to non-instrumented fusion) in patients older than 50 years. We observed the same tendency of better outcome in those patients who achieved a solid fusion as did Kornblum et al. We did, however, only use plain radiographs for fusion assessment; thus, it is likely that the fusion rate is overestimated—as it has been shown to be reduced by the use of more detailed diagnostic modalities (Brodsky et al. 1991). However, the uncertainty in determining fusion rate does not affect our main observation, which was the difference in functional outcome between the two groups. Other documentation for any relation between outcome and achievment of solid fusion has been somewhat controversial. In a meta-analysis, Mardjetko et al. (1994) could not demonstrate any relationship between fusion rates and patient satisfaction. In a historical study on pedicle screw fixation, Yuan et al. (1994) found higher fusion rates and better outcomes in patients fused with pedicle screw instrumentation than in patients with uninstrumented fusions.

What argued against the use of instrumentation was the higher number of additional spine surgeries in this group, as additional spine surgery after the primary procedure was associated with poorer outcome. Several studies have investigated the rate of complications associated with spinal surgery in this age group (Deyo et al. 1992, Carreon et al. 2003, Ragab et al. 2003, Cassinelli et al. 2007), but few have related the presence of complications or additional surgeries to functional outcome. In the Maine lumbar spine study, additional spine surgery over an 8–10-year follow-up period was associated with smaller improvement and less satisfaction as compared to those who had only undergone the primary intervention, which, however, rarely involved fusion but only decompression (Atlas et al. 2005). Tokuhashi et al. (2008) reported a high degree of independence 10 years after instrumented fusion in patients over 70 years. They did not, however, report on the influence of complications on outcome. In a study similar to ours, Glassman et al. (2007) reported inferior results in patients older than 65 years who required revision surgery after a primary lumbar fusion, as compared to patients only operated once. Despite the higher number of additional spinal surgeries in the instrumented group, outcome was still better than in the non-instrumented group. Thus, the poorer results associated with additional surgery could not outweigh the better outcome achieved in the instrumented group in general.

One study investigating the long-term results of decompressive surgery has shown a deterioration in improvement with time (Jonsson et al. 1997). In fusion surgery, the stability of the improvement in outcome achieved has varied between studies (Ekman et al. 2005, Andersen et al. 2008). In the current study, the improvement in both groups was stable and the long-term effect of the fusion procedure appears to be preserved also in this patient category.

In summary, we have found that superior outcomes can be achieved in selected patients over 60 years of age who have been treated with instrumented spinal fusion using allograft, as compared to non-instrumented fusion. The study suggests that the achievement of a solid fusion was one of the explanatory factors for this finding. However, pedicle screw instrumentation was associated with a larger number of additional surgeries, which resulted in inferior outcomes. Thus, the selection of procedure for the older patient requiring spinal fusion remains a balancing act, but instrumentation should not be discarded just because of the age of the patient. Future research should concentrate on determining the most efficient fusion procedure in elderly patients.
